# Universality in spectral condensation

**DOI:** 10.1038/s41598-020-73956-7

**Published:** 2020-10-15

**Authors:** Induja Pavithran, Vishnu R. Unni, Alan J. Varghese, D. Premraj, R. I. Sujith, C. Vijayan, Abhishek Saha, Norbert Marwan, Jürgen Kurths

**Affiliations:** 1 Department of Physics, Indian Institute of Technology Madras, Chennai, 600036 India; 2 Department of Mechanical and Aerospace Engineering, University of California San Diego, San Diego, CA 92093 USA; 3 Department of Aerospace Engineering, Indian Institute of Technology Madras, Chennai, 600036 India; 4 Potsdam Institute for Climate Impact Research, 14473 Potsdam, Germany; 5 Department of Physics, Humboldt University, 12489 Berlin, Germany; 6 Institute for Complex Systems and Mathematical Biology, University of Aberdeen, Aberdeen, AB 24 UK

**Keywords:** Physics, Statistical physics, thermodynamics and nonlinear dynamics

## Abstract

Self-organization is the spontaneous formation of spatial, temporal, or spatiotemporal patterns in complex systems far from equilibrium. During such self-organization, energy distributed in a broadband of frequencies gets condensed into a dominant mode, analogous to a condensation phenomenon. We call this phenomenon spectral condensation and study its occurrence in fluid mechanical, optical and electronic systems. We define a set of spectral measures to quantify this condensation spanning several dynamical systems. Further, we uncover an inverse power law behaviour of spectral measures with the power corresponding to the dominant peak in the power spectrum in all the aforementioned systems.

## Introduction

During self-organization, an ordered pattern emerges from an initially disordered state. In dynamical systems, a pattern can be any regularly repeating arrangements in space, time or both^1^. For example, a laser emits random wave tracks like a lamp until the critical pump power, above which the laser emits light as a single coherent wave track with high-intensity^2^. A macroscopic change is observed in the laser system as a long-range pattern emerges in time. Another example is the Rayleigh–Bénard system. For lower temperature gradients, the fluid parcels move randomly. As the temperature gradient is increased, a rolling motion sets in and the fluid parcels behave coherently to form spatially extended patterns. The initial random pattern can be regarded as a superposition of a variety of oscillatory modes and eventually some oscillatory modes dominate, resulting in the emergence of a spatio-temporal pattern^3,4^. Self-organization often results in the redistribution of energy from a wide range of frequencies to a few dominant modes to form periodic patterns. Such a condensation in the spectrum is analogous to the condensation phenomenon observed in classical and quantum systems, and we call this phenomenon spectral condensation.

Bose-Einstein condensation (BEC) occurring in quantum systems is characterized by occupation of the same energy level by a large fraction of the particles as temperature approaches absolute zero^5,6^. The transition to the condensate state, where the particles act collectively as a wave, can be viewed as the emergence of an ordered pattern from a disordered state of particles having different energy. Researchers have reported the observation of light condensation with the emission spectrum collapsing to the frequency of the lowest-loss mode^7–10^. Similarly, by drawing parallels to BEC, condensation phenomenon has been used to explain several dynamical transitions where an ordered final state is achieved from an initially disordered state even in classical systems^11^. For instance, a population of coupled oscillators forms a dynamical condensate where the condensation phenomenon leads to global synchronization among the group of oscillators^12^. Likewise, the framework of BEC has been utilized in predicting the competitive dynamics in the evolution of complex networks^13.^.

During self-organization driven by positive feedback leading to an ordered state in fluid mechanical, optical or electronic systems, we observe spectral condensation in the power spectrum of the appropriate system variables (in the emission spectrum for the optical system). While pattern formation in such systems has been studied extensively, universal characteristics of pattern formation or condensation in these systems garnered less attention. In conditions where the system is influenced by external noise or inherent fluctuations, the emergence of such a periodic pattern can be gradual as the parameter is varied. In this study, we quantify spectral condensation across various systems by defining a set of spectral measures based on the power spectrum. The power of the dominant mode is found to scale with these spectral measures following an inverse power law. From experimental observations, we find that systems exhibiting self-organization driven by positive feedback follow a unique way of spectral condensation in spite of different underlying physical mechanisms. Note that the self-organization that we are discussing in this paper is different^14^ from the concept of self-organized criticality (SOC) introduced by Per Bak and collaborators^15,16^.

## Results

**Figure 1:**
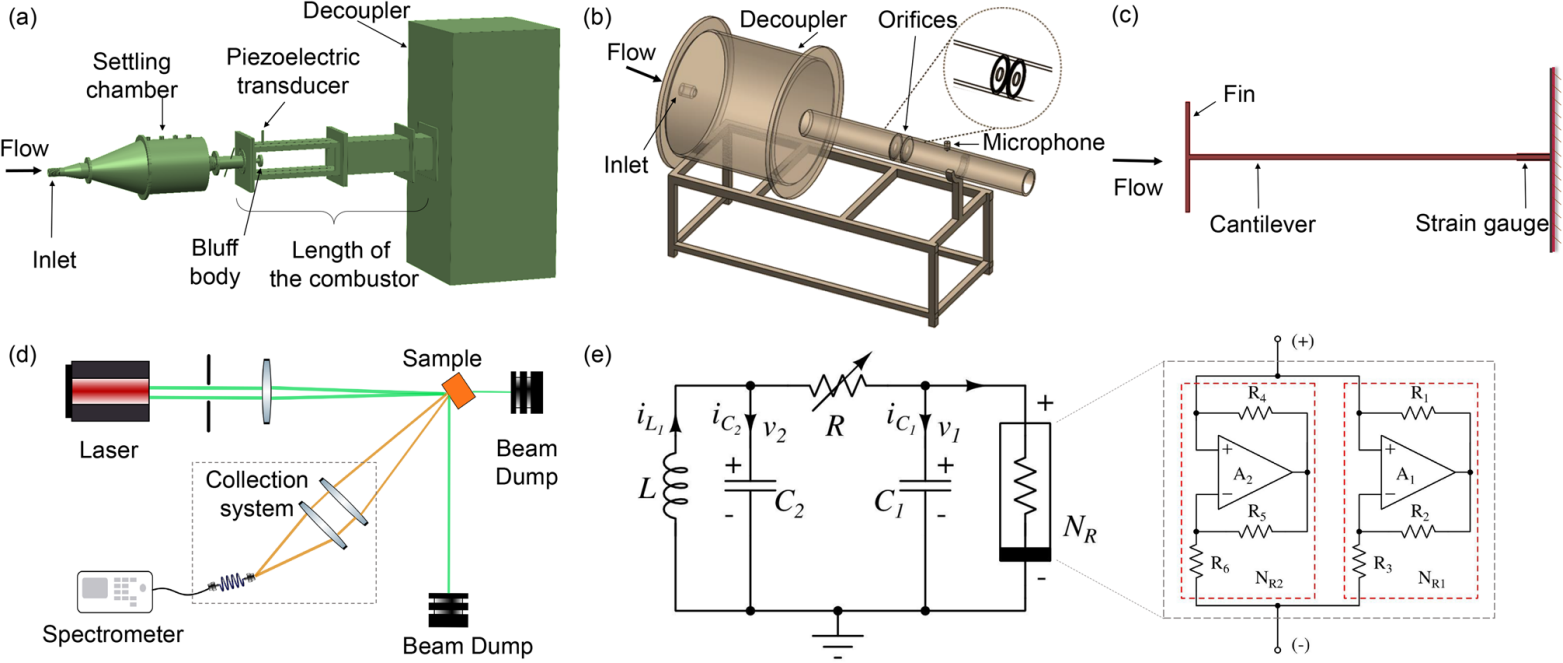
Schematic of the experimental setups. (**a–c**) Three fluid mechanical systems exhibiting oscillatory instabilities, namely, a thermoacoustic, an aeroacoustic and an aeroelastic system respectively. In these fluid mechanical systems, the Reynolds number (*Re*) is the control parameter. (**d**) An optical system for random lasing, wherein we observe a transition towards narrow-band lasing like emission as we increase the excitation pulse energy (EPE) of the laser source. (**e**) Electronic circuit showing Chua’s circuit along with a zoomed detailed view of the Chua’s diode. The variable resistor (*R*) is varied to obtain the transition from a fixed point to limit cycle oscillations. In all these systems, we acquire data for different values of the respective control parameters. We measure the acoustic pressure fluctuations for thermoacoustic and aeroacoustic systems while the strain on the cantilever beam is acquired for the aeroelastic system. For the random laser, the output emission is collected using a fibre optic spectrometer and the voltage ($v_1$) is measured for the electronic circuit. The dimensions of different experimental setups are not to scale.

Fluid mechanical systems examined in this study include thermoacoustic, aeroacoustic and aeroelastic systems which exhibit transition to oscillatory instabilities upon varying a control parameter. In a thermoacoustic system, the positive feedback between the acoustic field and the reactive flow field inside a confinement can cause an emergence of coherent dynamics in the flow field, which manifests as large amplitude self-sustained oscillations in pressure and heat release rate. This dynamical state is known as thermoacoustic instability^17^. The large amplitude oscillations encountered are detrimental to the structural integrity of practical systems such as rockets and gas turbine engines^18,19^. Aeroacoustic instability is another oscillatory instability which arises due to the interaction between the acoustic field and the vortices in a turbulent flow^20^. Aeroelastic instability occurs as a result of the coupling between the turbulent flow and the structural elements of the system^21^. Further details about the experiments are provided in Methods. We analyze the sharpening of the dominant peak in the power spectrum of a fluctuating system variable in the following cases: a thermoacoustic system with different flame holding mechanisms and different combustor lengths (Fig. 1a), an aeroacoustic system (Fig. 1b) and an aeroelastic system (Fig. 1c). Thermoacoustic system with different combustor lengths helps to achieve different characteristic time scales. Further, the two flame holding mechanisms in the combustor causes different mechanisms of thermoacoustic instability.

**Figure 2:**
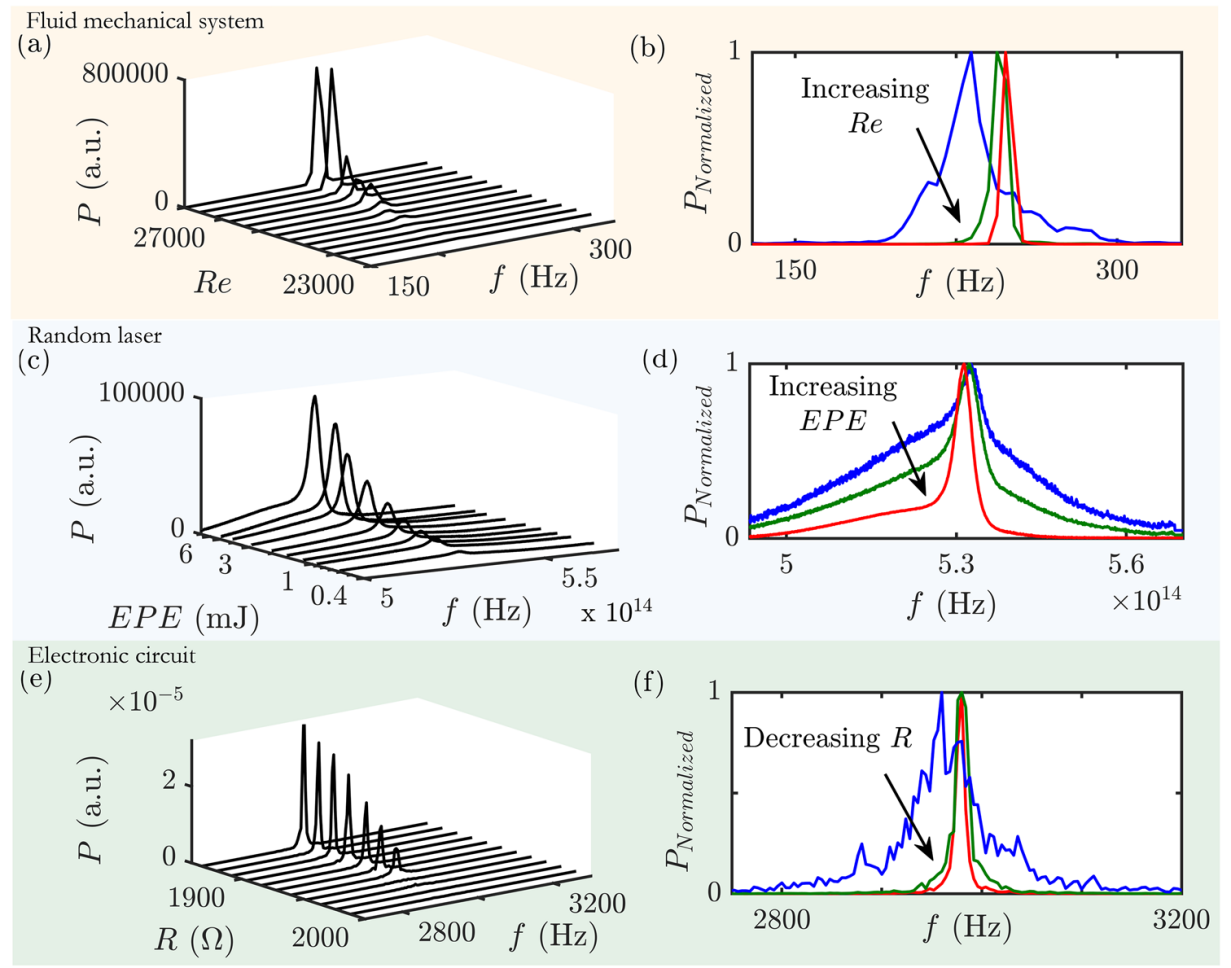
Evolution of the spectrum for fluid mechanical, optical and electronic systems. The evolution of power spectrum with variation in the corresponding control parameter is presented on the left side and the normalized spectra for each system are given on the right side of the panel. (**a,b**) Power spectra obtained using Fast Fourier Transform (FFT) of the acoustic pressure fluctuations for a laboratory-scale bluff body stabilized combustor of length 700 mm. The power spectra exhibit an increasing dominance of a single peak on approaching oscillatory instabilities (for increasing *Re*) in all the fluid mechanical systems discussed in this paper, and hence this figure is a representative example. (**c,d**) Emission spectra of the random laser as we progressively increase EPE. The power spectrum is obtained by multiplying the photon count per second for each wavelength with its respective energy. The broad spectrum starts to become a narrow lasing-like peak with increase in EPE. (**e,f**) Power spectra obtained using FFT of the voltage signal ($v_1$) measured from Chua’s circuit and the corresponding normalized spectra are shown respectively. The peak sharpens during the transition to limit cycle oscillations as the resistance (*R*) is decreased. The power spectra using FFT are plotted for a resolution of 4 Hz for visualization purpose.

All the three fluid mechanical systems exhibit a transition to oscillatory instability as we increase the Reynolds number (*Re*). Here, we present the evolution of the power spectrum only for a representative set of data (Fig. 2a), although all aforementioned cases of the fluid mechanical systems have been analyzed. The power spectrum has a broad peak for low values of *Re*. We observe the transition of power spectra from a broad peak to a sharp one as we approach the onset of the oscillatory instability. Each spectrum is normalized with its maximum amplitude to emphasize the narrowing of the peak (Fig. 2b).

The optical system chosen for this study is a random lasing system (Fig. 1d). Unlike conventional lasers, the lasing action in random lasers is achieved by strong multiple scattering in the optical gain medium. The large number of scatterers which are dispersed in the gain medium causes the light rays to scatter multiple times before they exit the gain medium^22,23^. The emission spectrum of a random laser, upon excitation by a pulse of suitable wavelength, is acquired using a fibre optic spectrometer. There is an appreciable narrowing in the emission profile (Fig. 2c) with the increment in excitation pulse energy (EPE), as is evident in Fig. 2d where each spectrum is normalized with its maximum power.

To study spectral condensation in electronic systems, we select Chua’s circuit (Fig. 1e) which has become a paradigm for chaos^24,25^. It consists of two capacitors, an inductor, a resistor and one nonlinear element known as Chua’s diode. The system exhibits period-doubling bifurcation from a fixed point to chaos with change in the resistance, *R*. Here, we focus on the transition from a fixed point to a period-1 limit cycle. In experiments, external noise or inherent fluctuations including thermal fluctuations of the electronic devices, their inaccuracies and electromagnetic interference will make the fixed point noisy^26^. Thus, for the conditions for which a fixed point is expected, we observe low amplitude noisy oscillations with a broad peak in the power spectrum centered around the natural frequency. During this transition to limit cycle (noisy Hopf bifurcation), we find a narrowing of the power spectrum (Fig. 2e,f) akin to that observed in fluid mechanical and optical systems.

Next, we quantify the sharpening of the power spectrum during spectral condensation by defining ‘spectral measures’. The general expression for the spectral measure is:

1 $$\begin{aligned}{}[\mu _m^x\ \mu _n^y]=\left[ \int _{-\delta F}^{+\delta F} \frac{P(F)}{P_0}\left| \frac{F}{f_{0}}\right| ^{m} d F\right] ^{x} \times \left[ \int _{-\delta F}^{+\delta F} \frac{P(F)}{P_0}\left| \frac{F}{f_{0}}\right| ^{n} d F\right] ^{y}. \end{aligned}$$

Here, $\mu _m$ is the $m^{th}$ moment of the power spectrum. *P*(*F*) represents the power corresponding to the modified frequency $F = f - f_0$, where *f* is a variable indicating the frequency of oscillations, $f_0$ is the frequency corresponding to the dominant peak in the power spectrum, and $P_0 = P(f_0)$. The indices $m,\ n,\ x$ & $\ y$ of the spectral measure are chosen to be positive integers. As our interest is to study the condensation towards a single peak, we compute the spectral measures $[\mu _m^x \ \mu _n^y]$ only in the neighbourhood of width $\delta F$ centered at $f_0$. We set $\delta F$ to $f_{0} / 5$, based on our analysis of a collection of data with vastly different values of $f_0$. Also, the amplitude of the peak reduces significantly within this range. Variations in the choice of $\delta F$ can be tried out, based on the appearance of the spectrum, such that it covers the spread of the peak during condensation. The spectral measures, $[\mu _m^x \ \mu _n^y]$ can be considered as the products of moments of the power spectrum raised to integer powers. According to the definition of the spectral measures, $[\mu _m^x \ \mu _n^y]$ decreases as the peak gets sharper. In this study, we present the analysis of three representative spectral measures, $[\mu _2]$, $[\mu _2 \ \mu _0]$ & $[\mu _4 \ \mu _4]$. Note that $[\mu _2]$ is the second moment of the power spectrum in the $\delta F$ neighbourhood of $f_0$, whereas, $[\mu _2 \ \mu _0]$ & $[\mu _4 \ \mu _4]$ are the products of higher moments of the distribution. Higher moments give more weightage to the tail ends of the spectrum and thus its variation indicates how the broad tails diminish.

**Figure 3:**
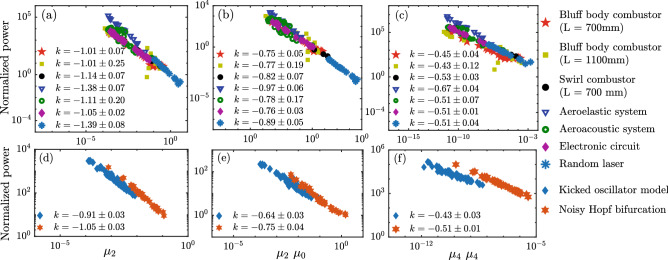
The scaling behaviour of spectral measures with the peak power during spectral condensation in experimental systems and models. Variation of the normalized power ($P_0/P_N$) corresponding to the dominant mode with the representative spectral measures ($[\mu _2]$, $[\mu _2 \ \mu _0]$ & $[\mu _4 \ \mu _4]$) plotted in double logarithmic scale **(a-c)** for the data acquired from experiments conducted in different systems and (**d-f**) for the data generated from two models. The extent of spectral condensation and the peak power differs by orders of magnitude across these systems. Hence, we rescale the power corresponding to the dominant peak ($P_0$) as $P_0/P_N$ to show the lines, $\log (P_0) = k\ \log \left( [\mu _m^x\ \mu _n^y]\right) + C$, in the same plot. The normalization factor, $P_N$, is the estimated value of peak power for $[\mu _m^x\ \mu _n^y] = 1$ obtained by extrapolating the line $\log (P_0) = k\ \log \left( [\mu _m^x\ \mu _n^y]\right) + C$ for each system. This choice of $P_N$ forces all lines to have $C=0$. We observe an inverse power law behaviour for all the spectral measures in the experiments as well as in the models. The uncertainties in the power law exponent are shown for $95\%$ confidence intervals.

We uncover an inverse power law relation between the spectral measures and the power corresponding to the dominant peak (Fig. 3a–c) during spectral condensation. All the data sets for the fluid mechanical, the optical and the electronic systems collapse to an inverse power law scaling in spite of the different physics involved in the process of condensation. We also present the analysis of data obtained from two models: kicked oscillator model^27^ and noisy Hopf bifurcation model^28^ (detailed descriptions are given in Methods). Both the models exhibit a transition from low amplitude aperiodic oscillations to a high amplitude limit cycle, thereby a condensation behaviour is observed in the power spectrum. We detect a similar scaling relation between $[\mu _m^x\ \mu _n^y]$ and $P_0/P_N$ (Fig. 3d–f) as observed in experiments. This inverse power law behaviour appears to be a universal characteristic of spectral condensation and the experimentally observed value for the power law exponent (*k*) corresponding to the spectral measures $[\mu _2]$, $[\mu _2 \ \mu _0]$ & $[\mu _4 \ \mu _4]$) are around $-1.12 \pm 0.13$, $-0.7 \pm 0.08$ and $-0.50 \pm 0.06$ respectively (averaged across systems). The exponent (*k*) is found to reduce for the higher indices of the measure and *k* for higher moments have much narrower dispersion across different systems (refer Methods). It may be noted that we do not ignore any points as power law tails and do not introduce any cutoffs in the power law scaling.

The existence of multiple invariant exponents motivates us to think about the existence of a universal form for the power spectrum in the neighbourhood of $f_0$. The power law relations indicate that given a distribution of power over a range of frequencies, the spectral measures at all levels of spectral condensation is already determined by the inverse power law relations. Further, the power spectrum decays away from $f_0$ and this decay is steeper for a sharp peak with higher amplitude. Thus, we consider a functional form for the power spectrum which is a function of *F* and has $P_0$ and $f_0$ as two parameters, and is as follows:

2 $$\begin{aligned} P(F) = P_{0} e^{\left[ -\left( P_{0}\right) ^{\alpha }\left( \frac{F}{f_{0}}\right) ^{\beta }\right] }, \end{aligned}$$

where all the symbols retain their definitions. Here, both $\alpha$ and $\beta$ have to be strictly positive. By comparing with the experimentally obtained values of power law exponents (*k*) for a set of spectral measures (for combinations of $m,\ n,\ x$ & *y*), we estimate the optimal values of the parameters $\alpha$ and $\beta$ iteratively as $\alpha = 0.125 \pm 0.017$ and $\beta = 0.317 \pm 0.024$ respectively.

## Discussion

In this paper, we define spectral measures to compare and quantify spectral condensation in different systems and we uncover a universal route through which spectral condensation occurs in fluid mechanical, optical and electronic systems. The dominant peak in the power spectrum sharpens with an increase in peak power following inverse power law relations with the spectral measures. Interestingly, the scaling exponents are found to be within a small range across all the systems studied. In addition, we note that the area under the curve in the power spectrum is not constant during the process of spectral condensation. It is not merely a redistribution of energy in the power spectrum; there is amplification due to transfer of energy across subsystems through the positive feedback mechanism. Thus, the total power associated with the system variable is not conserved during spectral condensation.

From a practical viewpoint, these spectral measures can be used as a system independent method to quantify dynamical transitions in systems where an emergent periodic behaviour is observed. During spectral condensation in fluid mechanical systems, the condensation of power to a dominant mode causes high amplitude periodic oscillations which can have catastrophic effects on the system. In such cases estimating the peak power during oscillatory instability will help to design control strategies to mitigate oscillatory instabilities^29^. In future studies, it will be interesting to study this scaling behaviour for biological systems.

## Methods

### Experiments

We perform experiments in three fluid mechanical systems (thermoacoustic, aeroacoustic and aeroelastic systems), an optical system (random laser) and an electronic system (Chua’s circuit). Details of all the experiments are provided below (refer Fig. 1).

#### Thermoacoustic system

The experimental setup comprises a settling chamber, ignition spark plug, combustion chamber equipped with a flame holding mechanism and a decoupler. Air partially premixed with fuel (Liquified petroleum gas, LPG: butane 60$\%$ and propane 40$\%$ composition by mass) enters through the inlet and the reactant mixture gets ignited just before it enters the combustion chamber. Two flame stabilizing mechanisms (bluff body and swirl) are used in the combustor which results in completely different flow physics with different mechanisms causing thermoacoustic instability^30,31^. In order to achieve different acoustic length scales and timescales, we vary the length of the combustion chamber. Travel time of sound in the duct is determined by the ratio of length of the duct to the speed of sound. We use two lengths (700 mm and 1400 mm) of bluff-body stabilized combustor and a swirl stabilized one a length 700 mm. Detailed descriptions of all the configurations of experiments can be found in Nair & Sujith^32^, Nair *et al*.^33^ and Unni & Sujith^34^ and the data analyzed in this study are reported in the above references.

We vary *Re* by increasing the mass flow rate of air and keeping the mass flow rate of the fuel constant. The mass flow rates of both air and fuel are controlled using mass flow controllers (Alicat MCR series) with an uncertainty of 0.8$\%$ of reading + 0.2$\%$ of full scale. The variation of *Re* is from ($1.81 \pm 0.052$) $\times 10^4$ to ($2.8 \pm 0.073$) $\times 10^4$ for the bluff body stabilized combustor of length 700 mm. For bluff body stabilized combustor of length 1400 mm, *Re* is varied from (1.96 ± 0.006) $\times 10^4$ to (3.53 ± 0.099)$\times 10^4$. Similarly, *Re* is varied from (1.61 ± 0.041)$\times 10^4$ to (1.96 ± 0.060) $\times 10^4$ for the swirl stabilized combustor. The system transitions towards limit cycle oscillations from a state of aperiodic oscillations with increase in *Re*. A piezoelectric transducer PCB106B50 transducer with a sensitivity of 72.5 mV/kPa and resolution 0.48 Pa is used for measuring the unsteady pressure fluctuations inside the combustion chamber for the configuration having a combustor length of 700 mm. A PCB103B02 transducer with a sensitivity of 217.5 mV/kPa and resolution 0.15 Pa is used for the combustor with length 1400 mm. In order to record the maximum amplitude of the standing wave, the sensor is mounted at the antinode of the acoustic oscillations. The sampling rate of data acquisition is 10 kHz for the experiments in the thermoacoustic system.

#### Aeroacoustic system

The aeroacoustic system consists of circular orifices in a duct. The flow passing through an orifice sheds vortices downstream. A feedback mechanism between the vortex shedding and the acoustic field inside the duct can generate tonal sounds under certain conditions. Periodic vortex shedding can excite the natural acoustic modes of the duct^35^. Figure 1b shows a schematic of the aeroacoustic experimental setup. The turbulent flow enters through a large cylindrical chamber, called the decoupler, which helps to maintain the ambient pressure at both ends of the duct by isolating the duct from the upstream pressure fluctuations. Inside the long pipe, there are two circular orifices of diameter 20 mm each, thickness 2.5 mm and separated by a distance of 18 mm. The length of the pipe before the first orifice and after the second orifice are 300 mm and 225 mm respectively. The mass flow rate of air is increased to attain different dynamical states in the system. A mass flow controller (Alicat MCR series) with an uncertainty of 0.8$\%$ of reading + 0.2$\%$ of full scale is used to control the mass flow rates. The mass flow rate of the air in the duct is increased from 1.633 ± 0.054 g/s to 2.695 ± 0.062 g/s in steps of 0.041 g/s. Thus, the *Re* varies from 5615 ± 185 to 9270 ± 212. We use a pressure field pre-polarized condenser microphone and preamplifier system (PCB make, model number 378C10, 1 mV/Pa sensitivity and 28.3 Pa resolution) to measure the pressure fluctuations inside the duct. The sensor is mounted at a distance of 100 mm from the second orifice. The data is acquired at a sampling rate of 10 kHz.

#### Aeroelastic system

The aeroelastic system is made up of a cantilever beam (length = 45 mm, width = 25 mm and thickness = 0.5 mm) with one end fixed and the other end free. The cantilever has a 12 mm long vertical fin at the free end of the beam, analogous to the winglet of an aircraft wing. A turbulent jet of air is passed from left to right along the length of the cantilever. The vortices shed from the edges of the fins induce unsteady aerodynamic load to the cantilever beam forcing it to oscillate. A strain gauge (Micro measurements make, pattern: 125LW, 3$\%$ strain range) is attached at a distance of 5 mm from the fixed end of the beam to acquire the resulting strain on the cantilever beam. We vary *Re* from 2384 ± 159 to 4768 ± 111 in order to capture the transition towards aeroelastic instability.

#### Optical system

A random lasing system consists of an optical gain medium with a large number of scatterers dispersed in a random arrangement^22^. The gain medium emits light upon excitation by a suitable wavelength. Analogous to the standard cavity mirrors, the feedback obtained through appropriate multiple scattering scenarios accounts for the amplification. We perform random lasing experiments with zinc oxide particles (ZnO) as scatterers and Rhodamine 6G dye as the gain medium. 50 mg of ZnO powder and 1 mg of Rhodamine 6G dye are dissolved in 3 ml of ethylene glycol to make a colloid and is filled in a quartz cuvette for the experiment. Figure 1d represents the experimental setup for the random lasing. We excite the sample with the second harmonic of an Nd:YAG laser source ($\lambda$ = 532 nm) with a repetition rate of 10 Hz and a pulse duration of 120 ps. The sample is positioned at an acute angle ($50^o \pm 0.5^o$) to the incident beam and the emission is captured by the collection lenses from the side. This arrangement helps to avoid the reflected rays of the excitation source from the cuvette. We increase EPE to achieve lasing. The output emission is collected by the tip of the fiber optic cable. The emission spectrum is obtained using Research India RIS-T1708 spectrometer.

#### Electronic circuit

Chua’s circuit is used to study spectral condensation during the transition from a fixed point to a limit cycle (Fig 1e shows the schematic of the circuit). It is a third-order, autonomous electronic circuit having a linear resistor, two linear capacitors, a linear inductor and one nonlinear element, known as Chua’s diode^25,36^. Chua’s diode has a nonlinear (piecewise-linear) $v - i$ characteristics^36^. More details and diagram can be found in Kennedy^37^. The circuit parameters are chosen to be $C_1$ = 10 nF, $C_2$ = 100 nF and L = 18 mH. By reducing the variable resistor, *R* from 2000 $\Omega$ towards 0 $\Omega$, Chua’s circuit exhibits a sequence of bifurcations. Here, we vary the control parameter *R* to observe a transition from a fixed point to limit cycle oscillations. The voltage ($v_1$) is acquired using a 16 bit A/D card (NI6343) at a sampling rate of 200 kHz.

### Mathematical models

#### Kicked oscillator model

We use a kicked oscillator model to study the scaling observed during the transition to limit cycle oscillations. Fluid mechanical systems that involve vortex shedding can be modelled as kicked oscillators^27^. In the model, the kicks are random when we are far from the self-organized state and become periodic as we approach the onset of oscillatory instability. In lasers, we observe a similar behaviour. The individual electrons vibrate and emit light randomly until it reaches a threshold excitation power and suddenly all of them start to vibrate uniformly in phase, forming a giant wave track^2^. The order parameter, *i.e.*, the wave generated by electron by their uniform vibration, forces every electron to vibrate in phase, leading to a typical synergetic phenomenon.

The kicked oscillator model captures the state of aperiodic oscillations, intermittency and limit cycle oscillations as observed in practical systems mentioned earlier. Seshadri *et al.*^27^ used a Galerkin expansion to express the acoustic variables in terms of the natural modes of the duct. They obtained a kicked oscillator equation for the acoustics modes as follows:

3 $$\begin{aligned} \ddot{\eta }_{n}+\xi _{n} {\dot{\eta }}_{n}+\omega _{n}^{2} \eta _{n}=B_{n} \sum _{j} \delta \left( t-t_{j}\right) \end{aligned}$$

Here $B_n$ is the kicking strength, $t_j$s known as the kicking times are the time instants of each kick. The kicking time instants $t_j$s are defined as follows:

4 $$\begin{aligned} t_{j}=t_{j-1}+\left( 1-C\left( p_{a}\right) \right) T_{a}+ C\left( p_{a}\right) \sigma T_{a}|{\mathscr {N}}(0,1)| \end{aligned}$$

Here $T_a$ is the dominant time period, ${\mathscr {N}}$ (0, 1) is the Gaussian white noise and $\sigma$ is the strength of the noise. *C* is a biased coin toss where 1 occurs with probability $p_a$ and 0 with a probability $1-p_a$ and $p_a$ is called the aperiodic probability. $p_a$ is an estimate of the aperiodic content in the time series. Here, we can think of the aperiodic probability as a parameter (like the Reynolds number) that we are varying to achieve the different dynamical states of the system. From Eqs. (3) and (4) we can observe that for $p_a = 0$, the kicks are periodic and hence the dynamics correspond to a state of limit cycle oscillations. Whereas $p_a = 1$, the kicks occur at random time instants and hence this corresponds to a state of aperiodic oscillations.

Following Seshadri et al.^27^, the kicking strength *B* is kept constant as 100 for this study and the noise strength $\sigma$ is selected as 5 and 1 for the aperiodic case and limit cycle, respectively. We vary the aperiodic probability from 1 to 0 (corresponding to aperiodic fluctuations to limit cycle) in steps of 0.001 to obtain time series corresponding to each aperiodic probability. Now for each of these time series, the Fourier transform was evaluated and the spectral measures are computed. Now the variation of spectral measures and the power corresponding to the dominant mode of oscillations are plotted (Fig. 3d-f). We can see that this model captures the inverse power law relationship that was observed in the experimental data.

#### Noisy Hopf bifurcation model

We examine another model of a nonlinear oscillator in the presence of noise which exhibits subcritical and supercritical Hopf bifurcation.

5 $$\begin{aligned} \ddot{\eta }+\alpha {\dot{\eta }}+\omega ^{2} \eta ={\dot{\eta }}\left( \beta +K \eta ^{2}-\gamma \eta ^{4}\right) +\xi \end{aligned}$$

Here, $\alpha$ and $\beta$ are linear damping and driving respectively. Following Noiray^28^, we use additive white noise $\xi$ of intensity $\Gamma$ and having an autocorrelation $<\xi \xi _{\tau }>= \Gamma \delta (\tau )$. The values of the parameters $\omega$, $\beta$, $\gamma$, *K* and $\Gamma$ are kept constant ($\omega$ = 2$\pi \times$ 120 rad/s, $\beta$ = 50 rad/s, $\gamma$ = 0.7, *K* = 9, $\Gamma = 10^5$). The linear damping ($\alpha$) is varied from 95 rad/s to 56 rad/s to capture the transition from an aperiodic state to a high amplitude limit cycle oscillation. The corresponding time series of $\eta$ for each value of $\alpha$ is obtained and the spectral measures are evaluated from the power spectra of the time series of $\eta$. As observed for the experiments, the spectral measures follow inverse powerlaw scaling with peak power for noisy Hopf bifurcation as well, where periodicity arises out of a noisy environment.

### Power law exponents for a set of spectral measures

**Figure 4:**
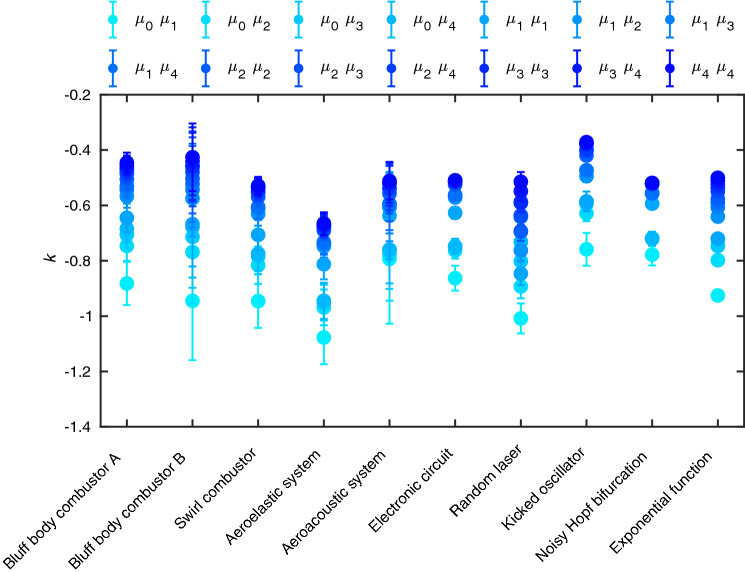
Power law exponents obtained from the scaling relation between the spectral measures and the peak power for the higher index spectral measures in different experimental systems and models. Here, the exponential function refers to the functional form of the power spectrum as defined in Eq. (2) in the manuscript.

The set of spectral measures show an inverse power law relation with the power corresponding to the dominant mode during spectral condensation. The power law exponents decrease for the higher indices of the measure. The power law exponents calculated for different indices up to *m* = 4 and *n* = 4 are summarized in Fig. 4. We have compared the exponents for different experimental systems and models. In Fig. 4, bluff body combustor A and B represent bluff body combustor with length 700 mm and 1100 mm respectively.
